# Combined use of metformin and atorvastatin attenuates atherosclerosis in rabbits fed a high-cholesterol diet

**DOI:** 10.1038/s41598-017-02080-w

**Published:** 2017-05-19

**Authors:** Fei Luo, Yuan Guo, Gui-yun Ruan, Jun-ke Long, Xi-long Zheng, Qin Xia, Shui-ping Zhao, Dao-quan Peng, Zhen-fei Fang, Xiang-ping Li

**Affiliations:** 1 Department of Cardiovascular Medicine, The Second Xiangya Hospital, Central South University, Changsha, Hunan 410011 China; 2 Smooth Muscle Research Group, Department of Biochemistry & Molecular Biology, Libin Cardiovascular Institute of Alberta, Faculty of Medicine, University of Calgary, Alberta, Canada; 3 Department of Orthopedics, The Second Xiangya Hospital, Central South University, Changsha, Hunan 410011 China

## Abstract

Statins are widely used to reduce cardiovascular risk. Unfortunately, some patients still experience cardiovascular events though prescribed with high-intensity statins. Metformin, an anti-diabetic drug, was reported to possess anti-atherosclerotic effects. Therefore, the experiments were designed to evaluate whether combined use of metformin and atorvastatin can achieve additional benefits. In rabbits fed a high-cholesterol diet, we evaluated the effects of the combination therapy on atherosclerotic plaques, lipid profiles, blood glucose levels, liver and kidney functions. Effects of combination therapy on cholesterol efflux and the expression of related transporters were studied *in vitro*. Our results showed that the combination therapy induced a more significant decrease in atherosclerotic lesion area than atorvastatin without additional lipid-lowering effect. The combination therapy significantly increased the percentage of large high-density lipoprotein subfraction. The intravenous glucose tolerance test showed that atorvastatin-treated rabbits had an increased area under the curve for time-dependent glucose levels after a bolus injection of glucose, which was completely reversed by metformin treatment. In cultured macrophages, co-treatment with metformin and atorvastatin promoted cholesterol efflux and up-regulated expression of ATP-binding cassette transporters A1 and G1. Taken together, our results suggest that atorvastatin/metformin combination therapy may achieve additional anti-atherosclerotic benefits likely through increasing cholesterol efflux in macrophages.

## Introduction

Atherosclerotic cardiovascular disease (ASCVD) is a leading cause of mortality and morbidity worldwide. Several large-scale clinical trials have shown that statins significantly reduced cardiovascular events in patients with ASCVD or at high risk, which was mainly ascribed to their lipid-lowing effects^1, 2^. To date, statins have been recommended as the first-choice drugs for prevention and treatment of ASCVD by clinical guidelines^3^. However, a significant proportion of statin-treated patients remains at elevated risk of cardiovascular events, which has been referred to as “residual risk”^4, 5^. Besides, statins increased hemoglobin A1c (HbA1c) levels as well as the risk of new-onset diabetes in a dose-dependent manner^6, 7^. Some combinational therapies, such as statin plus ezetimibe, have been found better than statin monotherapy^8^. Thus, it is critical to developing new strategies to reduce the residual risk and improve safety in statin-treated patients.

Metformin, a first-line medication for the treatment of type 2 diabetes (T2D), was reported to improve insulin sensitivity and reduce HbA1c levels in statin-treated patients with or without impaired fasting glucose levels^9, 10^. Metformin also has anti-atherosclerotic properties beyond its main glucose-lowering effect^11, 12^. In a randomized controlled trial, metformin treatment for 3 years was associated with a substantial reduction in major cardiovascular events in patients with T2D and coronary artery disease (CAD)^12^. Preclinical studies also revealed an anti-atherosclerotic effect of metformin^13, 14^. However, it is still unknown whether the combination of metformin and statins has an add-on effect on atherosclerosis and how it affects lipid profiles and glucose metabolism. We speculate that metformin in combination with statins may enhance the anti-atherosclerotic effect and counteract some of the unwanted adverse effects of statin.

Our study was designed to evaluate the effectiveness and safety of an atorvastatin/metformin combination therapy in a rabbit atherosclerosis model induced by a high-cholesterol diet. The potential mechanisms of this combination therapy were also explored.

## Results

### Atorvastatin/metformin combination therapy attenuates atherosclerotic plaques more effectively than monotherapy

**Figure 1:**
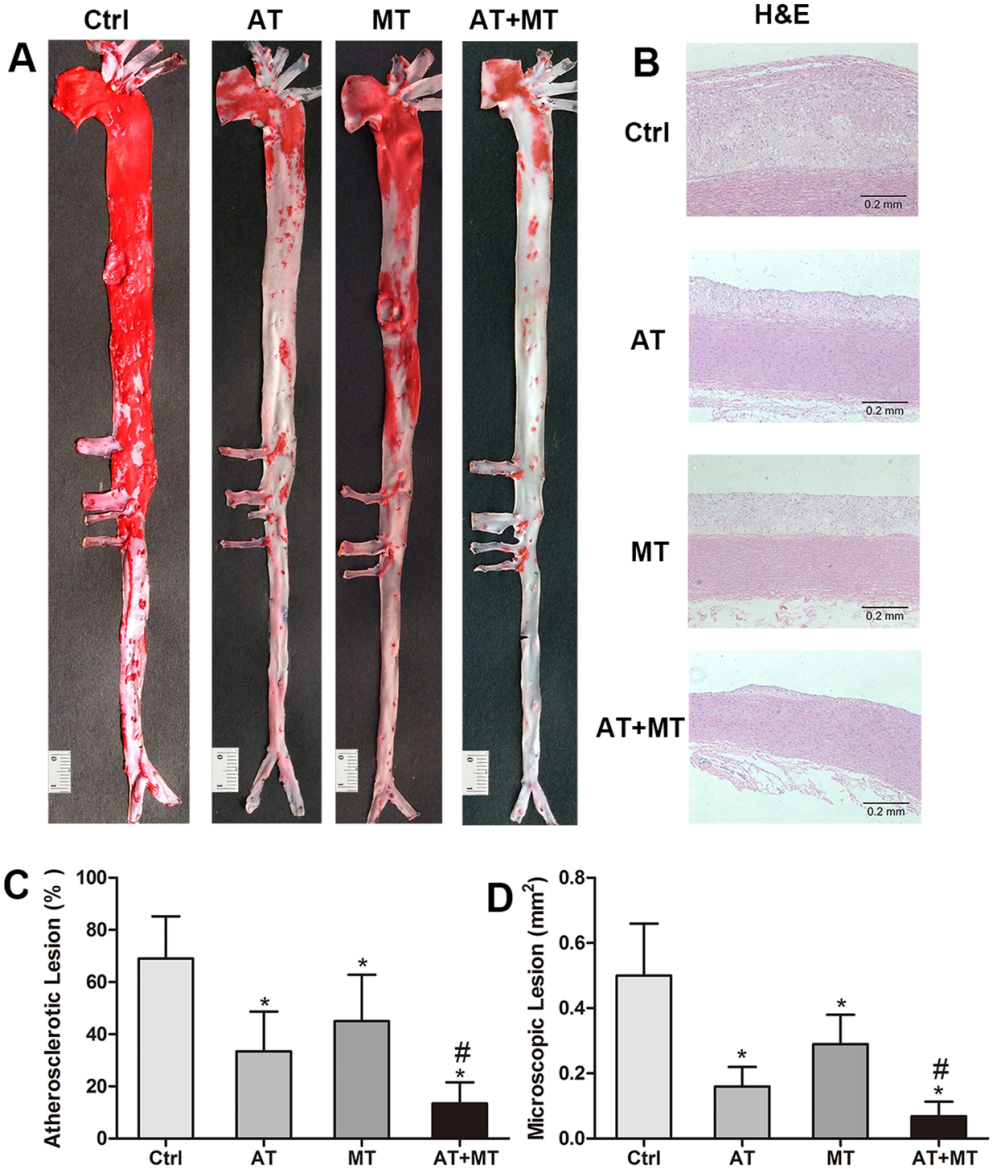
Atorvastatin/metformin combination therapy attenuates atherosclerotic plaques more effectively than monotherapy with either medication. At the end of the experiment, aortas between the aortic arch and the junction of the iliac arteries of rabbits were separated and stained with Oil Red O (**A**). To analyze microscopic atherosclerotic lesion, sections from the aortic arch were cut for paraffin sections and stained with H&E (**B**). Gross en face atherosclerotic lesion area of the whole aorta (**C**) and microscopic lesion area (**D**) were quantified by ImageJ software. Data are mean ± SD, n = 6 or 7 for each group. *P < 0.05, compared with Ctrl group; ^#^ *P* < 0.05, compared with AT group. Ctrl, control group; AT, atorvastatin group; MT, metformin group; AT + MT group, atorvastatin/metformin combination therapy group.

As shown in Fig. 1A and C, a 12-week high-cholesterol diet induced a significant increase in atherosclerotic lesion area in rabbits in the control (Ctrl) group; after 10 weeks of atorvastatin or metformin treatment, the atherosclerotic lesion area was significantly reduced by 51% and 35%, respectively. Atorvastatin/metformin combination therapy resulted in an 80% reduction of atherosclerotic plaques compared with the Ctrl group, which was more effectively than each monotherapy (all *P* < 0.05). Microscopic quantification of lesion area was also measured in aortic arch sections. Compared with Ctrl group, the treatment of atorvastatin or metformin significantly reduced the lesion size by 68% and 42%, respectively, while atorvastatin/metformin combination therapy further reduced atherosclerotic lesion size by 86% (all *P* < 0.05) (Fig. 1B and D).

### Metformin added to atorvastatin therapy has no additional lipid-lowering effect

**Figure 2:**
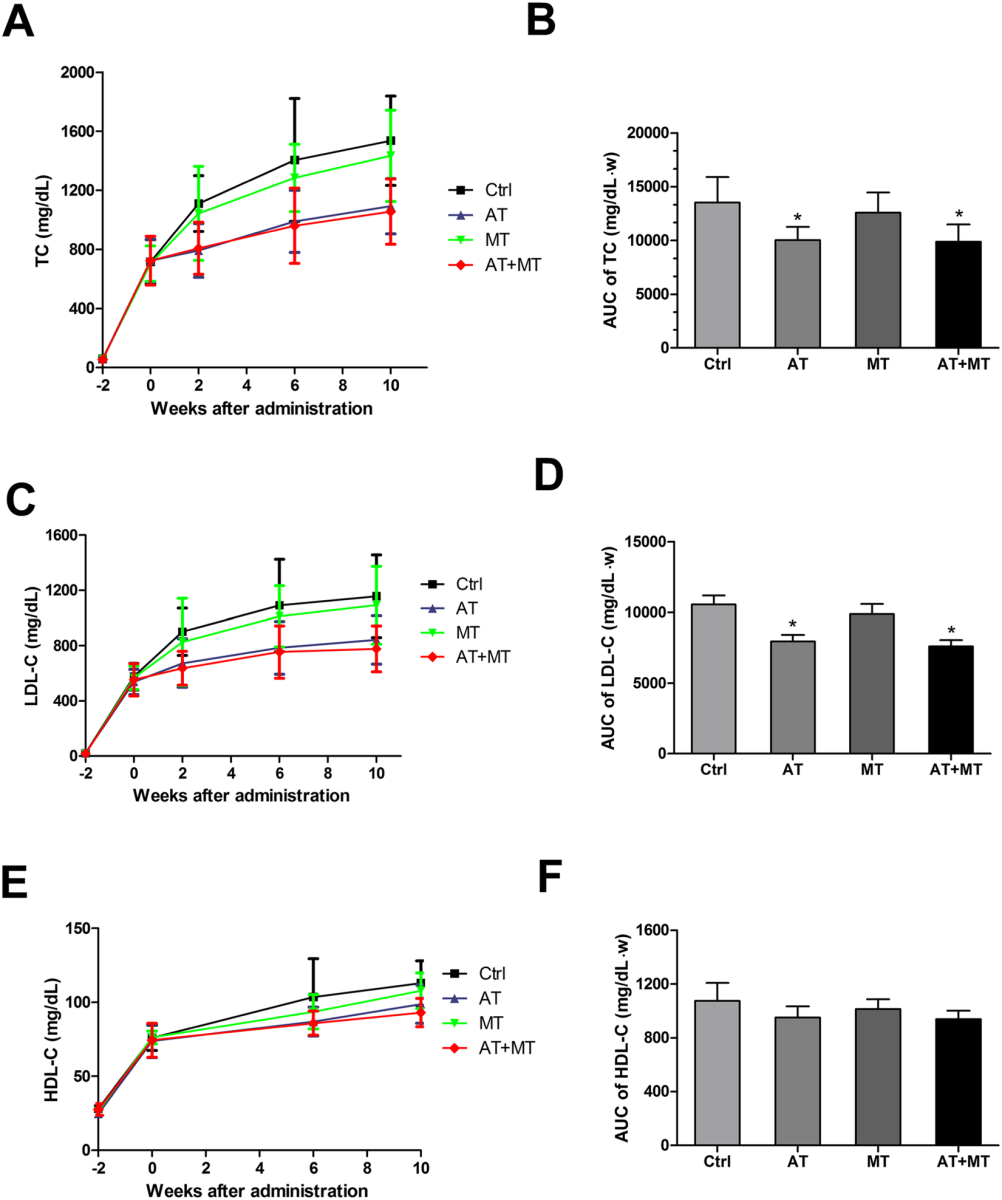
Metformin added to atorvastatin therapy has no additional lipid-lowering effect. At the beginning of the experiment, rabbits were fed a high-cholesterol diet. After 2 weeks, rabbits were randomly stratified into the normal sodium (Ctrl group), atorvastatin (AT group), metformin (MT group), or atorvastatin/metformin combination therapy (AT + MT group) for a period of 10 weeks. Lipid levels were measured at −2, 0, 2, 6, 10 weeks after drug administration. Time-dependent changes and their AUC values of serum TC levels (**A**,**B**), LDL-C levels (**C**,**D**) and HDL-C levels (**E**,**F**) are presented. Data are mean ± SD, n = 7 for each group. *P < 0.05, compared with Ctrl group. AUC, area under the curve; HDL-C, high-density lipoprotein cholesterol; LDL-C, low-density lipoprotein cholesterol; TC, total cholesterol.

After 12-week high-cholesterol diet, the serum TC in the Ctrl group increased from 59 ± 10 mg/dL to 1536 ± 302 mg/dL, while LDL-C levels increased from 19 ± 8 mg/dL to 1156 ± 327 mg/dL, and HDL-C levels increased from 28 ± 5.35 mg/dL to 113 ± 39.9 mg/dL (all *P* < 0.05) (Fig. 2A,C and E). After 10-week treatment with metformin (MT), the serum concentrations of TC and LDL-C in MT group were slightly decreased without a significant difference when compared with the Ctrl group (*P* = 0.36 and *P* = 0.41) (Fig. 2B and D). Atorvastatin treatment significantly decreased serum concentrations of TC by 26% and LDL-C by 25%, while atorvastatin/metformin combination therapy decreased the concentrations of TC and LDL-C by 27% and 28%, respectively, compared with the Ctrl group (all *P* < 0.05) (Fig. 2B and D). There was no significant difference in the lipid-lowering effects of atorvastatin monotherapy and atorvastatin/metformin combination therapy (all *P* > 0.05). Metformin, atorvastatin or the combination therapy with atorvastatin/metformin did not significantly affect serum concentrations of HDL-C (Fig. 2F) or triglyceride (TG) (data not shown).

### Metformin added to atorvastatin therapy increases serum large HDL subfractions

It is well known that HDL plays a pivotal role in cholesterol efflux. It was reported that large HDL is inversely associated with cardiovascular disease^15^. As shown in Table 1, treatment with metformin and the combination therapy increased the percentage of large HDL compared with Ctrl group (all *P* < 0.05). Atorvastatin treatment did not significantly affect the percentage of large HDL.

**Table1:** HDL subfractions at 10 weeks after administration.

Subfractions	Ctrl	AT	MT	AT + MT
Large	66.0 ± 9.7%	69.7 ± 10.1%	86.9 ± 7.1%^*,#^	81.7 ± 7.7%^*,#^
Moderate	30.3 ± 2.6%	24.4 ± 8.5%	11.7 ± 7.2%^*,#^	15.6 ± 6.4%^*,#^
Small	3.7 ± 8.2%	5.9 ± 7.8%	1.4 ± 3.8%	2.7 ± 6.0%

Percentage of HDL subfractions at 10 weeks after administration is expressed as mean ± SD. *P < 0.05 compared with Ctrl group; ^#^ *P* < 0.05, compared with AT group. Ctrl, control group; AT, atorvastatin group; MT, metformin group; AT + MT group, atorvastatin/metformin combination therapy group.

### Metformin added to atorvastatin therapy is well tolerated and partially reverses atorvastatin-induced adverse effects

**Figure 3:**
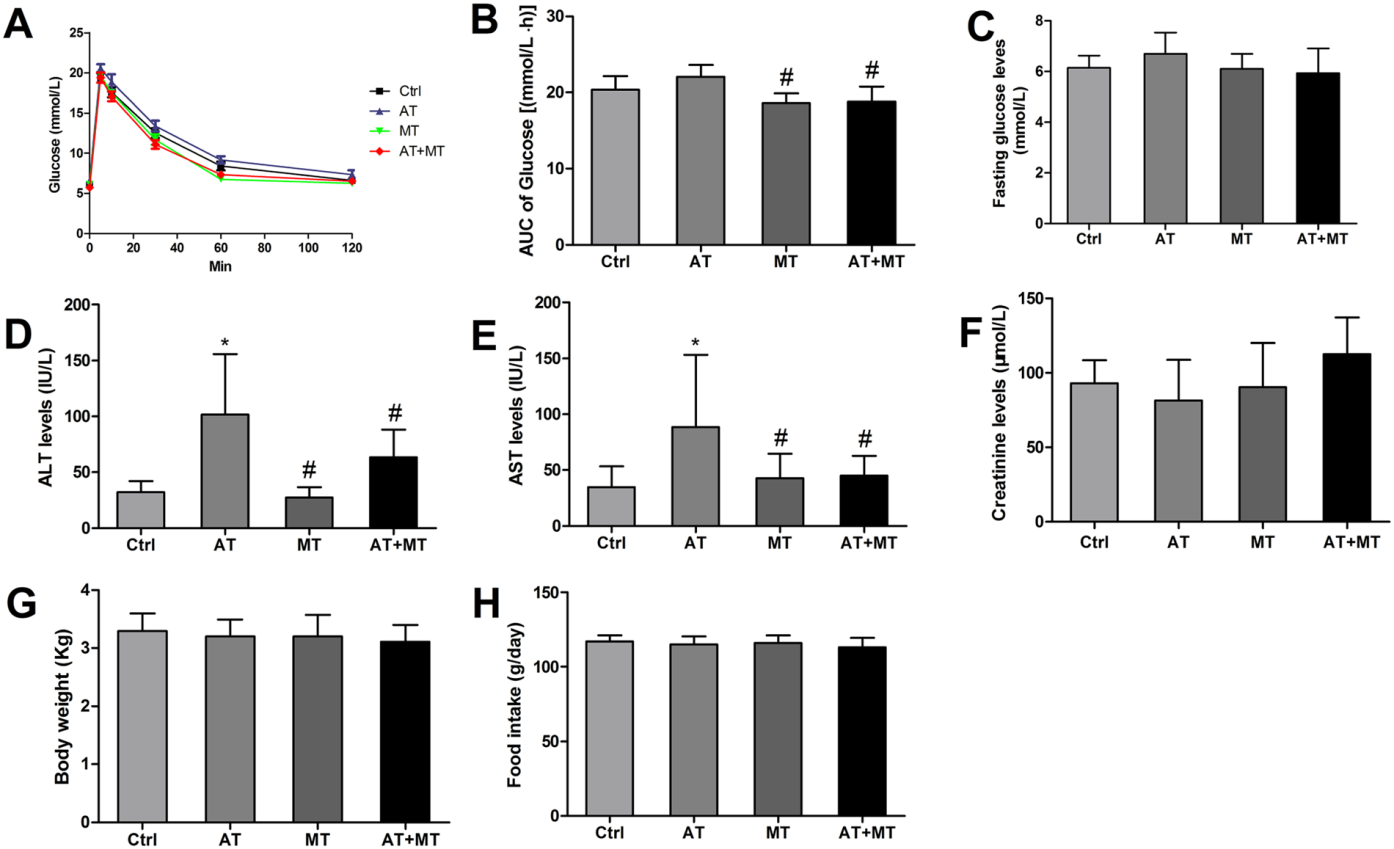
Metformin added to atorvastatin therapy is well tolerated and partially reversed atorvastatin-induced adverse effects. At 10 weeks after medication administration, the intravenous glucose tolerance test was performed. Briefly, rabbits were fasted overnight, then a bolus of glucose (0.6 g/kg body weight) was injected into the ear vein, and blood samples were collected at 0, 5, 10, 30, 60, and 120 min. Time-dependent changes in plasma glucose levels (**A**) and their AUC values (**B**) are presented. Fasting plasma glucose levels (**C**), serum concentrations of ALT (**D**), AST (**E**) and creatinine (**F**), body weight (**G**) and food intake (**H**) were also measured at 10 weeks after medication administration. Data are mean ± SD, n = 7 for each group. *P < 0.05, compared with Ctrl group; ^#^ *P* < 0.05, compared with AT group. Ctrl, control group; AT, atorvastatin group; MT, metformin group; AT + MT group, atorvastatin/metformin combination therapy group. ALT: alanine transaminase; AST: aspartate transaminase; AUC, area under the curve.

The intravenous glucose tolerance test (IVGTT) was performed in rabbits at the end of treatments. The blood glucose concentrations were measured at 0, 5, 15, 30, 60 and 120 min after a bolus injection of glucose, and the changes in serum concentrations of glucose were presented in Fig. 3A. When compared with Ctrl group, the incremental area under the curve (AUC) for glucose from rabbits in the AT group was higher than that in Ctrl group (Fig. 3B), but the difference was not statistically significant (*P* = 0.08). However, the AUC for glucose from rabbits in the MT or AT + MT group was significantly lower than that of the atorvastatin group (*P* < 0.05) (Fig. 3B).

Metformin, atorvastatin, and their combination therapy had no effect on fasting serum glucose levels (Fig. 3C), serum creatinine levels (Fig. 3F), body weight (Fig. 3G) or food intake (Fig. 3H). Metformin did not affect serum concentrations of ALT and AST (Fig. 3D and E). Atorvastatin increased serum concentrations of ALT and AST compared with those in the Ctrl group (all *P* < 0.05), while atorvastatin/metformin combination therapy did not significantly increase the concentrations of ALT and AST (Fig. 3D and E).

### Mortality of rabbits

During the study, one rabbit died from gavage accident in the AT group and AT + MT group, respectively. One rabbit died from diarrhea in Ctrl group and MT group, respectively.

### Metformin promotes cholesterol efflux and expression of ABCA1 and ABCG1 in acLDL-loaded THP-1 macrophages

**Figure 4:**
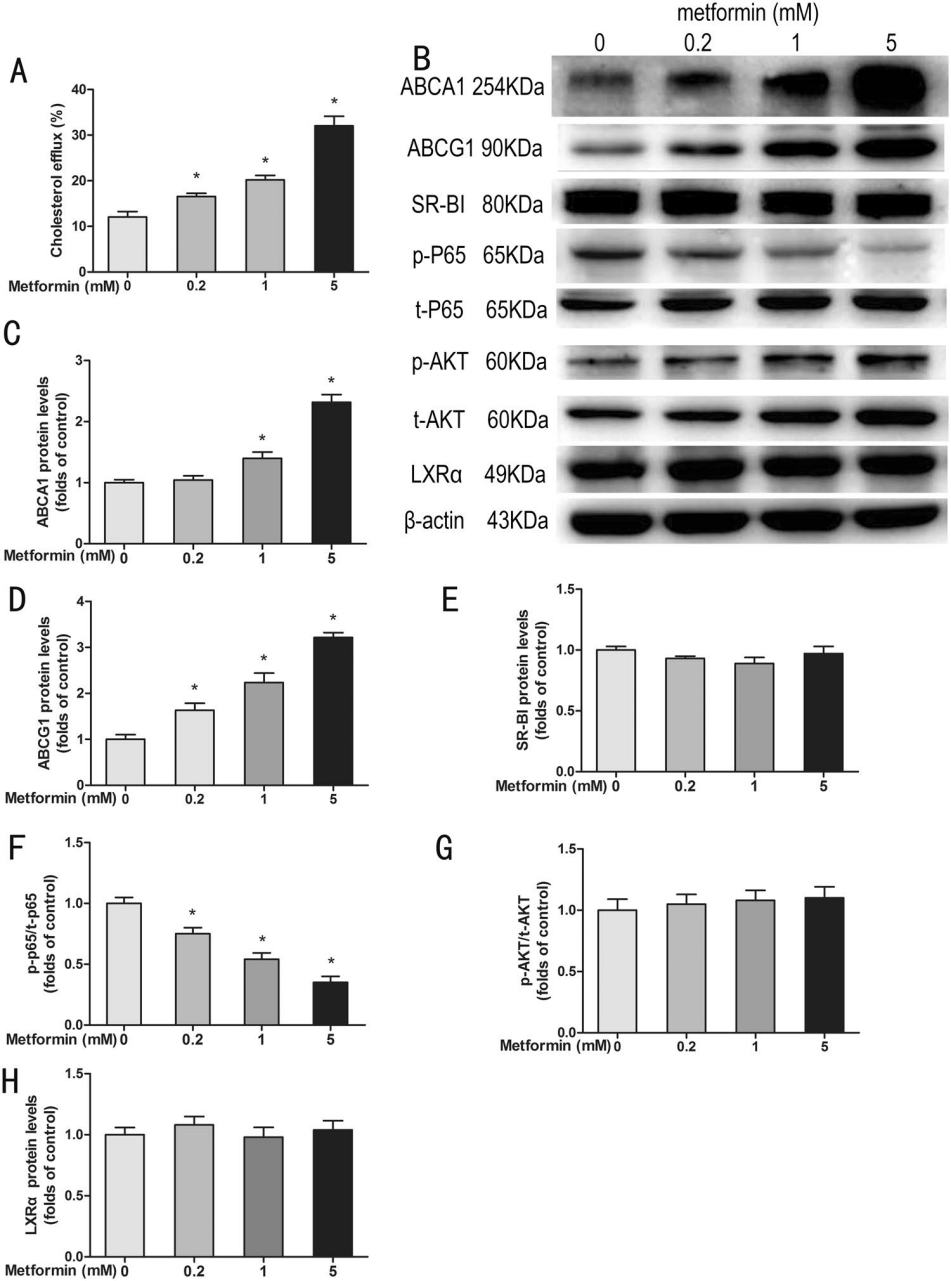
Metformin improves cholesterol efflux and ATP-binding cassette transporters expression *in vitro*. After loading with acLDL (50 μg/mL) and ^3^H-cholesterol (0.5 μCi/mL) for 24 h, THP-1 macrophages were incubated with 5% apoB-depleted serum and different concentrations of metformin (0, 0.2, 1 and 5 mmol/L) for a further 24 h. Cholesterol efflux was expressed as the percentage of radioactivity in the medium relative to the total quantity of radioactivity detected in the cells and medium (**A**). Examples of cholesterol efflux-related proteins tested by Western blot after 24 h metformin treatment (**B**). Relative protein levels of ABCA1 (**C**), ABCG1 (**D**), SR-BI (**E**), p-P65/t-P65 (**F**), p-AKT/t-AKT (**G**), and expression of LXRα (**H**). Data are mean ± SD, n = 3 for each group. *P < 0.05, compared with blank control group.

To explore the potential mechanism underlying the effects of combination therapy, we first examined whether metformin could improve cholesterol efflux. To do so, acLDL-loaded THP-1 macrophages were incubated with different concentrations of metformin (0, 0.2, 1 and 5 mmol/L) *in vitro* for 24 h in the presence of apoB-depleted serum. Our results showed that metformin significantly improved cholesterol efflux from macrophages into apoB-depleted serum in a concentration-dependent manner when compared with blank control (*P* < 0.05) (Fig. 4A).

It is well known that ATP-binding cassette transporters A1 (ABCA1) and G1 (ABCG1), and scavenger receptor class B type I (SR-BI) are key factors involved in macrophage cholesterol efflux^16^. Our further studies showed that metformin at the concentrations of 1 and 5 mmol/L, but not 0.2 mmol/L, up-regulated ABCA1 expression (*P* < 0.05) (Fig. 4B and C). Furthermore, metformin concentration-dependently increased ABCG1 expression (*P* < 0.05) (Fig. 4B and D), but not the expression of SR-BI (Fig. 4B and E). We then examined the involvement of upstream regulators of ABCA1 and ABCG1 expression, such as nuclear factor κB (NF-κB), liver X receptor α (LXRα), and AKT^17–20^. Our results showed that metformin did not affect LXRα expression or phosphorylation of AKT (Fig. 4B,G and H), but concentration-dependently inhibited phosphorylation of NF-κB p65 (*P* < 0.05) (Fig. 4B and F).

### Co-treatment with metformin and atorvastatin promotes cholesterol efflux and expression of ABCA1 and ABCG1 in acLDL-loaded THP-1 macrophages

**Figure 5:**
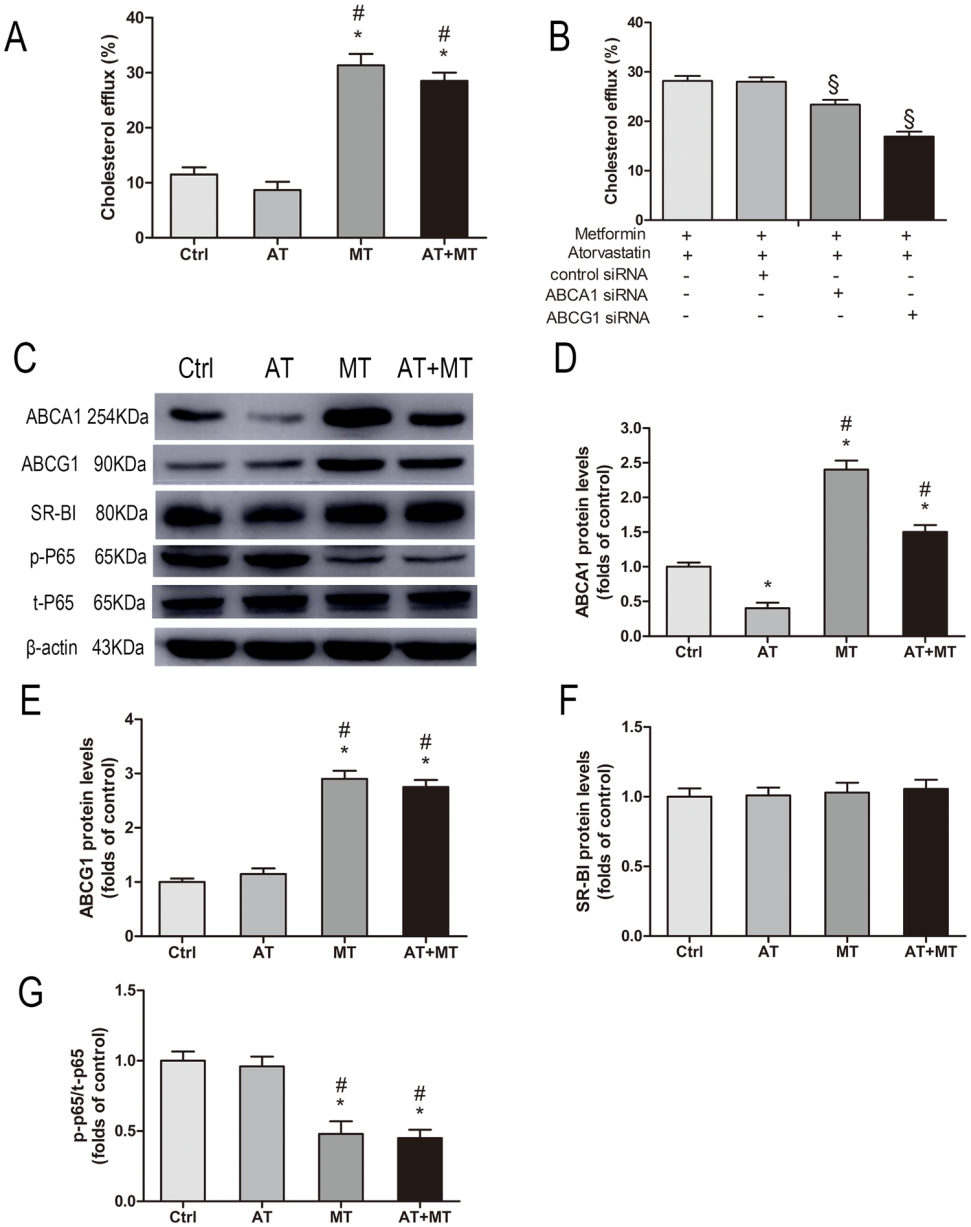
Co-treatment of metformin and atorvastatin promotes cholesterol efflux and expression of ABCA1 and ABCG1 in acLDL loaded THP-1 macrophages. After loading with acLDL (50 μg/mL) and ^3^H-cholesterol (0.5 μCi/mL) for 24 h, THP-1 macrophages were incubated with 5% apoB-depleted serum, metformin (5 mmol/L) and atorvastatin (10 μmol/L) for a further 24 h. Cholesterol efflux was expressed as the percentage of radioactivity in the medium relative to the total quantity of radioactivity detected in the cells and medium (**A**). Cells were transfected with control siRNA or siRNA-ABCA1/G1 and cholesterol efflux were measured (**B**). Examples of cholesterol trafficking regulators tested by Western blot (**C**). Relative protein levels of ABCA1 (**D**), ABCG1 (**E**), SR-BI (**F**), p-P65/t-P65 (**G**). Data are mean ± SD, n = 3 for each group. *P < 0.05, compared with Ctrl group; ^#^ *P* < 0.05, compared with AT group; ^§^ *P* < 0.05, compared with AT + MT + control siRNA group.

Previous studies showed that atorvastatin (at the concentration of 10 μmol/L) significantly reduced cholesterol efflux and expression of ABCA1 in human macrophages^21^. Therefore, we evaluated the effect of combined treatment with metformin (5 mmol/L) and atorvastatin (10 μmol/L) on cholesterol efflux and cholesterol trafficking regulators in acLDL-loaded THP-1 macrophages. Our results showed that co-treatment with metformin and atorvastatin significantly improved cholesterol efflux (*P* < 0.05), while treatment with atorvastatin alone had a trend to decrease cholesterol efflux (*P* = 0.07) (Fig. 5A). We then used siRNA approach to evaluating the role for ABCA1/G1 in cholesterol efflux. ABCA1 siRNA and ABCG1 siRNA were transfected into cells, respectively, before the combined treatment with metformin and atorvastatin. Our results showed that both siRNA-﻿ABCA1 and siRNA-ABCG1 induced significant reduction of cholesterol efflux, but the more significant reduction was observed for ABCG1 siRNA (all *P* < 0.05) (Fig. 5B). In addition, our results showed that the co-treatment with metformin and atorvastatin significantly up-regulated the expression of ABCA1 and ABCG1, and inhibited phosphorylation of NF-κB p65 (all *P* < 0.05) (Fig. 5C,D,E and G). Atorvastatin decreased the expression of ABCA1 (*P* < 0.05) but had no significant effects on the expression of ABCG1 or phosphorylation of NF-κB p65 (Fig. 5C,D,E and G). Neither atorvastatin nor metformin affected the expression of SR-BI (Fig. 5C and F).

## Discussion

In the current study, we used a high cholesterol diet-induced atherosclerotic model to evaluate effects of the combined use of atorvastatin and metformin. Our results suggest that atorvastatin and metformin combination therapy is superior to atorvastatin monotherapy for the treatment of atherosclerosis and the underlying mechanisms might be associated with cholesterol efflux in macrophages.

It is well known that statins attenuate atherosclerosis mainly through reducing LDL-C levels, but it is unclear how metformin exerts its anti-atherosclerotic effects. Our results have demonstrated that atorvastatin/metformin combination therapy did not exhibit a better lipid-lowering effect than atorvastatin, which is consistent with the recent clinical and preclinical data^10, 22^. The CAMERA study revealed that metformin did not affect the lipid profile in statin-treated patients^10^. Forouzandeh *et al*. found that metformin markedly reduced atherosclerotic plaques but did not affect plasma cholesterol in ﻿apoE^-/-^ ﻿mice fed a high-fat diet^13^. These data strongly suggest an additional anti-atherosclerotic mechanism for metformin when added to atorvastatin, which is independent of the lipid-lowering effect.

Our study is the first, to our knowledge, to demonstrate that atorvastatin/metformin combination therapy increases the percentage of large HDL subfraction. Goldberg *et al*.^23^ found that metformin could raise the concentrations of large HDL in a clinical trial. Previous research has suggested an inverse association of large HDL subfraction with coronary artery disease^24, 25^, which may involve reverse cholesterol transport (RCT). Notably, initiation and progression of atherosclerosis are known to involve multiple factors including dysfunction of RCT, dyslipidemia, microRNAs, and so on ref. 26. Cholesterol efflux from macrophages in aortic walls in response into HDL is an initial and important step of RCT. Small HDL accepts the transfer of cellular cholesterol and is then converted to large HDL^27^. Thus, an increase in large HDL possibly indicates its increased conversion, and large HDL may serve as a measure of the capacity for RCT. Therefore, our study indicated that atorvastatin/metformin combination therapy possibly increased cholesterol efflux. Cholesterol efflux from macrophages is mainly mediated by the ABCA1 and ABCG1^16^. Our further studies have revealed that metformin and atorvastatin have significant effects on cholesterol efflux and ABCA1/G1 expression in macrophages *in vitro*.

More specifically, metformin combined with atorvastatin promoted cholesterol efflux and the expression of ABCA1 and ABCG1 in acLDL-loaded THP-1 macrophages. Using ABCA1/G1 siRNA approach, we found that both ABCA1 and ABCG1 are involved in the increased cholesterol efflux induced by co-treatment of metformin and atorvastatin, in which ABCG1 may play a predominant role. In addition, we observed that atorvastatin decreased the expression of ABCA1 without affecting ABCG1 expression in acLDL-loaded THP-1 macrophages, keeping consistent with previous studies^21, 28^. Li *et al*.^29^ previously showed that metformin could increase ABCG1 expression in murine macrophages *in vitro*, suggesting that metformin may promote cholesterol efflux. Our current study indicated that atorvastatin/metformin combination therapy promoted cholesterol efflux from acLDL-loaded THP-1 macrophages into serum by up-regulating ABCA1 and ABCG1 expression. Increased cholesterol efflux into HDL may promote the conversion of small HDL to large HDL. Thus, the increased percentage of large HDL subfraction in AT + MT group may result from the up-regulation of cholesterol efflux induced by the combination therapy. Therefore, we conclude that the supplementary anti-atherosclerotic effect of metformin, when combined with atorvastatin, may be at least partially mediated by improving cholesterol efflux.

Our conclusion is further supported by the intracellular signaling mechanism. It is known that activation of NF-κB p65 and AKT suppresses ABCA1/ABCG1 expression, while activation of LXRα can up-regulate ABCA1/ABCG1 expression^17–20^. Our results showed that co-treatment with atorvastatin and metformin could inhibit phosphorylation of NF-κB p65 without altering phosphorylation of AKT and the expression of LXRα. Although the effect of co-administration of metformin and atorvastatin on NF-κB p65 was not reported before, metformin was reported to inhibit activation of NF-κB p65 in vascular wall *in vivo* ^14^ and inhibit phosphorylation of NF-κB p65 in smooth muscle cells *in vitro* ^30^.

In addition, our study showed that 2 mg/kg/day atorvastatin slightly increased the AUC for glucose levels after glucose loading, but there was no significant difference when compared with the Ctrl group. Cheng *et al*.^31^ observed a more significant delay in clearance of serum glucose induced by treatment with atorvastatin at approximately 50 mg/rabbit/day for 16 weeks. It was previously reported that statin-associated diabetes is both dose- and time-dependent^32, 33^. Importantly, our studies have shown that the delay in glucose clearance induced by atorvastatin was completely reversed by metformin. Supportively, Krysiak *et al*.^9^ indicated that metformin improved glucose metabolism of statin-treated patients with impaired fasting glucose. Therefore, the addition of metformin to atorvastatin therapy may counteract the adverse effect of atorvastatin on glucose metabolism. Our results of the safety profile for atorvastatin/metformin combination therapy suggest that atorvastatin/metformin combination therapy did not affect the function of the liver or kidneys, which is consistent with previous clinical studies^10^.

## Limitations

In this study, we demonstrated that addition of metformin to atorvastatin therapy enhances the anti-atherosclerotic effects in a rabbit model with a high-cholesterol diet. However, there are several limitations to our study. Although we demonstrated that metformin alone or combined with atorvastatin could improve cholesterol efflux by up-regulating ABCA1 and ABCG1 expression *in vitro*, we did not investigate whether such a phenomenon was replicated *in v*i*vo*. Besides, it is well known that endothelial cell function plays a pivotal role in atherosclerosis^34^. Santulli *et al*.^35, 36^ demonstrated that several miRNAs could modulate endothelial cell functions, which were important in diagnosis and treatment of atherosclerosis. In our study, we did not evaluate the endothelial functions and related miRNAs. Further studies are required to explore the potential signaling pathways *in vivo* and *in vitro* in order to provide robust evidence for this novel approach to anti-atherosclerotic therapy.

## Materials and Methods

### Atherosclerotic animal model

The protocol was approved by the Animal Research Committee, Central South University, Hunan, China and carried out in accordance with the Guidelines for Animal Experimentation of Central South University and the Guide for the Care and Use of Laboratory Animals published by the US National Institutes of Health (NIH Publication NO. 85-23, revised 2011).

Forty male New Zealand white rabbits (2.5 months, mean weight 2.3 ± 0.2 kg) were obtained from the Medical Experimental Animal Center of Hunan Province, China. Rabbits were housed in individual cages and standard environmental conditions. Rabbits were fed a high-cholesterol diet (0.5% cholesterol) of up to 120 g/day. After 2 weeks of the high-cholesterol diet, some rabbits were excluded because of their very high (>1000 mg/dL) or low (<500 mg/dL) serum total cholesterol (TC) concentrations. Animals were also excluded if they developed high alanine aminotransferase (ALT) values (>100 IU/L). The remaining rabbits were randomly assigned to four groups and exposed to the following interventions: control group (Ctrl group, n = 8), treated with normal saline; metformin group (MT group, n = 8), treated with 150 mg/kg/day metformin; atorvastatin group (AT group, n = 8), treated with 2 mg/kg/day atorvastatin; Atorvastatin/metformin combination group (AT + MT group, n = 8), treated with 150 mg/kg/day metformin and 2 mg/kg/day atorvastatin. Metformin hydrochloride tablets were purchased from Bristol–Myers Squibb (New York, NY, USA) and atorvastatin calcium was purchased from Pfizer (New York, NY, USA). Both of these were dissolved in normal saline and administrated by gastric gavage (orally) at a fixed time. The doses of the drugs were determined based on body surface area calculations of therapeutic human dosing (metformin of 2.5 g/day and atorvastatin of 40 mg/day). The rational to use a high cholesterol-fed rabbit model was that the lipid metabolism and pathologic characteristics of atherosclerosis in these animals more closely resemble those of humans compared with murine models^37^.

### Quantification of atherosclerotic lesions

At the end of the experiment, all rabbits were sacrificed by injection of an overdose of sodium pentobarbital solution. Between the aortic arch and the junctions of the iliac arteries, the aortas were separated from the surrounding tissues. From the initiation of the aortic arch, 0.5-cm sections were excised for paraffin treatment as previously described^38^. The remaining aortas were soaked in 4% paraformaldehyde and then stained with Oil Red O solution to evaluate the atherosclerotic lesion area of the aorta by image-processing software (ImageJ). For the microscopic quantification of lesions, a paraffin section from each rabbit was cut into at least 3 sections (80 μm) and stained with hematoxylin and eosin (H&E) before quantification using ImageJ software.

### Measurement of serum lipids and other biochemical markers

After animals were fasted for 16 h, blood samples were drawn from the middle artery of the ear and collected by coagulation-promoting tubes or EDTA anti-coagulant tubes. Blood samples were further collected at −2, 0, 2, 6 and 10 weeks after administration of medications. The concentrations of serum TC, LDL-C, HDL-C, TG, ALT, aspartate transaminase (AST), creatinine, and glucose were determined using enzymatic methods (bioMerieux, Lyon, France). ﻿HDL subfractions were mesured using Lipoprint HDL Subfractions Kit (Quantimetrix, Redondo Beach, CA, ﻿USA). ﻿﻿At the end of the experiment, IVGTT was performed. Rabbits were fasted overnight, and then a bolus of glucose (0.6 g/kg body weight) was injected into the ear vein, followed by collecting blood samples at 0, 5, 10, 30, 60, and 120 min.

### Human THP-1 cell culture and differentiation

Human THP-1 cells were obtained from the American Type Culture Collection (ATCC, Rockville, USA) and cultured as previously described^39^. For differentiation of monocytes to macrophages, THP-1 cells were seeded in 6-well plates (1 × 10^6^ cells per well) and treated with 100 ng/mL phorbol 12-myristate 13-acetate (PMA) (Sigma–Aldrich, St. Louis, USA) for 48 h.

### Cholesterol efflux *in vitro*

THP-1 macrophages were incubated with 50 μg/mL acetylated-LDL (acLDL) (Peking Union-Biology, Beijing, China) and loaded with 0.5 μCi/mL ^3^H-cholesterol for 24 h as previously described^40^. After loading, the cells were washed twice and then incubated in RPMI-1640 medium containing 5% (v/v) apoB-depleted serum with different concentrations of metformin (Selleck Chemicals, Houston, USA) or atorvastatin (Sigma–Aldrich, St. Louis, USA) for 24 h. Cholesterol efflux was measured by calculating the incorporation of ^3^H-cholesterol in the medium (extracellular radioactivity) and in the cells (intracellular radioactivity) after 10% NaOH extraction. Percentage efflux was calculated as follows: cholesterol efflux = extracellular cpm/(intracellular cpm + extracellular cpm) × 100%.

### Transfection with siRNA

SiRNA against ABCA1/G1 (siRNA-ABCA1/G1) was purchased from Santa Cruz Biotechnology, Inc (Santa Cruz, CA, USA). THP-1 macrophages were plated in 6-well plates and loaded with acLDL and ^3^H-cholesterol. Thereafter, control siRNA or siRNA-ABCA1/G1 was added to the respective well, followed by culture for 24 h and then metformin/atorvastatin were added to each well, and cholesterol efflux was mesured as described above.

### Western blotting analysis

The proteins from THP-1 macrophages were extracted with RIPA lysis buffer (Beyotime, Beijing, China) and separated on sodium dodecyl sulfate-polyacrylamide gel electrophoresis (SDS-PAGE) before transferring onto polyvinylidene difluoride (PVDF) membranes. Subsequently, the membranes were blocked with 5% milk PBST solution and then incubated with the following respective antibodies: mouse monoclonal anti-ABCA1, rabbit monoclonal anti-ABCG1, rabbit monoclonal anti-SR-BI (Abcam, Cambridge, USA), mouse monoclonal anti-NF-κB, rabbit polyclonal anti-phospho-NF-κB (Ser536), rabbit polyclonal anti-phospho-Akt (Ser473), rabbit polyclonal anti-Akt (CST, Danvers, USA), mouse monoclonal anti-LXR alpha (PPMX, Tokyo, Japan), or β-actin control (Bios, Beijing, China), at 4 °C for overnight. Blots were washed, incubated with the secondary antibody, and visualized by chemiluminescence.

### Statistical analysis

Data are presented as mean ± SD. Comparison of lipid and glucose levels between two independent groups was performed using the non-parametric Wilcoxon rank sum test. Comparisons between multiple groups were conducted using one-way analysis of variance (ANOVA) followed by least significant difference *post hoc* tests. The difference with *P* < 0.05 (two sides) was statistically significant. AUC was calculated according to the trapezium rule.

## References

[1] Baigent C (2005). Efficacy and safety of cholesterol-lowering treatment: prospective meta-analysis of data from 90,056 participants in 14 randomised trials of statins. Lancet.

[2] Cholesterol Treatment Trialists, C. *et al*. Efficacy and safety of more intensive lowering of LDL cholesterol: a meta-analysis of data from 170,000 participants in 26 randomised trials. *Lancet* **376**, 1670–1681, doi:10.1016/S0140-6736(10)61350-5 (2010). 10.1016/S0140-6736(10)61350-5 PMC2988224 21067804

[3] Stone NJ (2014). 2013 ACC/AHA guideline on the treatment of blood cholesterol to reduce atherosclerotic cardiovascular risk in adults: a report of the American College of Cardiology/American Heart Association Task Force on Practice Guidelines. Circulation.

[4] Kataoka Y (2015). Atheroma progression in hyporesponders to statin therapy. Arteriosclerosis, thrombosis, and vascular biology.

[5] Waters DD (2009). Lipid treatment assessment project 2: a multinational survey to evaluate the proportion of patients achieving low-density lipoprotein cholesterol goals. Circulation.

[6] Sattar N (2010). Statins and risk of incident diabetes: a collaborative meta-analysis of randomised statin trials. Lancet.

[7] Preiss D (2011). Risk of incident diabetes with intensive-dose compared with moderate-dose statin therapy: a meta-analysis. Jama.

[8] Cannon CP (2015). Ezetimibe Added to Statin Therapy after Acute Coronary Syndromes. N Engl J Med.

[9] Krysiak R, Okopien B (2013). The effect of metformin on monocyte secretory function in simvastatin-treated patients with impaired fasting glucose. Metabolism: clinical and experimental.

[10] Preiss D (2014). Metformin for non-diabetic patients with coronary heart disease (the CAMERA study): a randomised controlled trial. The lancet. Diabetes & endocrinology.

[11] Luo F, Guo Y, Ruan G, Li X (2016). Metformin promotes cholesterol efflux in macrophages by up-regulating FGF21 expression: a novel anti-atherosclerotic mechanism. Lipids in health and disease.

[12] Hong J (2013). Effects of metformin versus glipizide on cardiovascular outcomes in patients with type 2 diabetes and coronary artery disease. Diabetes care.

[13] Forouzandeh F (2014). Metformin beyond diabetes: pleiotropic benefits of metformin in attenuation of atherosclerosis. Journal of the American Heart Association.

[14] Li SN (2009). Metformin inhibits nuclear factor kappaB activation and decreases serum high-sensitivity C-reactive protein level in experimental atherogenesis of rabbits. Heart and vessels.

[15] Mora S (2009). Lipoprotein particle profiles by nuclear magnetic resonance compared with standard lipids and apolipoproteins in predicting incident cardiovascular disease in women. Circulation.

[16] Rosenson RS (2012). Cholesterol efflux and atheroprotection: advancing the concept of reverse cholesterol transport. Circulation.

[17] Zhao GJ (2014). NF-kappaB suppresses the expression of ATP-binding cassette transporter A1/G1 by regulating SREBP-2 and miR-33a in mice. International journal of cardiology.

[18] Zhao GJ (2013). Antagonism of betulinic acid on LPS-mediated inhibition of ABCA1 and cholesterol efflux through inhibiting nuclear factor-kappaB signaling pathway and miR-33 expression. PloS one.

[19] Dong F, Mo Z, Eid W, Courtney KC, Zha X (2014). Akt inhibition promotes ABCA1-mediated cholesterol efflux to ApoA-I through suppressing mTORC1. PloS one.

[20] Joseph SB (2002). Synthetic LXR ligand inhibits the development of atherosclerosis in mice. Proceedings of the National Academy of Sciences of the United States of America.

[21] Wang W, Song W, Wang Y, Chen L, Yan X (2013). HMG-CoA reductase inhibitors, simvastatin and atorvastatin, downregulate ABCG1-mediated cholesterol efflux in human macrophages. Journal of cardiovascular pharmacology.

[22] Kooy A (2009). Long-term effects of metformin on metabolism and microvascular and macrovascular disease in patients with type 2 diabetes mellitus. Archives of internal medicine.

[23] Goldberg R (2013). Lifestyle and metformin treatment favorably influence lipoprotein subfraction distribution in the Diabetes Prevention Program. The Journal of clinical endocrinology and metabolism.

[24] Xu RX (2015). High-density lipoprotein subfractions in relation with the severity of coronary artery disease: A Gensini score assessment. Journal of clinical lipidology.

[25] El Harchaoui K (2009). High-density lipoprotein particle size and concentration and coronary risk. Annals of internal medicine.

[26] Novak J, Olejnickova V, Tkacova N, Santulli G (2015). Mechanistic Role of MicroRNAs in Coupling Lipid Metabolism and Atherosclerosis. Advances in experimental medicine and biology.

[27] Colvin PL, Moriguchi E, Barrett PH, Parks JS, Rudel LL (1999). Small HDL particles containing two apoA-I molecules are precursors *in vivo* to medium and large HDL particles containing three and four apoA-I molecules in nonhuman primates. Journal of lipid research.

[28] Chen WM (2016). Modulation of microRNA Expression in Subjects with Metabolic Syndrome and Decrease of Cholesterol Efflux from Macrophages via microRNA-33-Mediated Attenuation of ATP-Binding Cassette Transporter A1 Expression by Statins. PloS one.

[29] Li D (2010). Adenosine monophosphate-activated protein kinase induces cholesterol efflux from macrophage-derived foam cells and alleviates atherosclerosis in apolipoprotein E-deficient mice. The Journal of biological chemistry.

[30] Isoda K (2006). Metformin inhibits proinflammatory responses and nuclear factor-kappaB in human vascular wall cells. Arteriosclerosis, thrombosis, and vascular biology.

[31] Cheng D (2015). Atorvastatin delays the glucose clearance rate in hypercholesterolemic rabbits. Biomedicine & pharmacotherapy = Biomedecine & pharmacotherapie.

[32] Cederberg H (2015). Increased risk of diabetes with statin treatment is associated with impaired insulin sensitivity and insulin secretion: a 6 year follow-up study of the METSIM cohort. Diabetologia.

[33] Wang KL (2014). Risk of new-onset diabetes mellitus versus reduction in cardiovascular events with statin therapy. The American journal of cardiology.

[34] Santulli G (2014). A selective microRNA-based strategy inhibits restenosis while preserving endothelial function. The Journal of clinical investigation.

[35] Santulli G (2016). MicroRNAs and Endothelial (Dys) Function. Journal of cellular physiology.

[36] Wronska A, Kurkowska-Jastrzebska I, Santulli G (2015). Application of microRNAs in diagnosis and treatment of cardiovascular disease. Acta physiologica.

[37] Fan J (2015). Rabbit models for the study of human atherosclerosis: from pathophysiological mechanisms to translational medicine. Pharmacology & therapeutics.

[38] Li Y (2014). Urotensin II promotes atherosclerosis in cholesterol-fed rabbits. PloS one.

[39] Vasamsetti SB (2015). Metformin inhibits monocyte-to-macrophage differentiation via AMPK-mediated inhibition of STAT3 activation: potential role in atherosclerosis. Diabetes.

[40] McGillicuddy FC (2009). Inflammation impairs reverse cholesterol transport *in vivo*. Circulation.

